# The origin of prostate gland-secreted IgA and IgG

**DOI:** 10.1038/s41598-017-16717-3

**Published:** 2017-11-28

**Authors:** Juliete A. F. Silva, Manoel F. Biancardi, Dagmar R. Stach-Machado, Leonardo O. Reis, Osvaldo A. Sant’Anna, Hernandes F. Carvalho

**Affiliations:** 1 Department Structural and Functional Biology, State University of Campinas, Campinas, Brazil; 2 Department Histology, Embriology and Cell Biology, Federal University of Goias, Goiânia, Brazil; 3 Center for Life Sciences, Pontificial University of Campinas, Campinas, Brazil; 4 Department Immunochemistry, Butantan Institute, São Paulo, Brazil; 5 National Institute of Science and Technology of Photonics Applied to Cell Biology (INFABiC), Campinas, Brazil

## Abstract

The prostate secretes immunoglobulin (Ig) A (IgA) and IgG; however, how immunoglobulins reach the secretion, where the plasma cells are located, whether immunoglobulins are antigen-specific and where activation of the adaptive response occurs are still unknown. Immune cells, including CD45RA^+^ cells, were scattered in the stroma and not organized mucosae-associated lymphoid-tissue. IgA (but not IgG) immunostaining identified stromal plasma cells and epithelial cells in non-immunized rats. Injected tetramethylrhodamine-IgA transcytosed the epithelium along with polymeric immunoglobulin receptor. Oral immunization with ovalbumin/mesopourous SBA-15 silica adjuvant resulted in more stromal CD45RA^+^/IgA^+^ cells, increased content of ovalbumin-specific IgA and IgG, and the appearance of intraepithelial CD45RA^+^/IgG^+^ cells. An increased number of dendritic cells that cooperate in other sites with transient immunocompetent lymphocytes, and the higher levels of interleukin-1β, interferon-γ and transforming growth factor-β, explain the levels of specific antibodies. Nasal immunization produced similar results except for the increase in dendritic cells. This immunomodulatory strategy seems useful to boost immunity against genitourinary infections and, perhaps, cancer.

## Introduction

Different mucosae secrete immunoglobulins. Both immunoglobulin (Ig)-A (dimers) and IgM (pentamers) are secreted by mucosal plasma cells in association with J chain. These Igs transcytose the epithelial layer after binding to the polymeric immunoglobulin receptor (pIgR) on the baso-lateral surface of epithelial cells. When exposed to the apical (luminal) surface of the epithelial cells, pIgR is proteolytically cleaved from the plasma membrane, releasing the secretory IgA (sIgA; a complex of the IgA, the J chain the secretory component of pIgR) and sIgM^1^. Current knowledge assumes that IgG does not complex with the J chain, does not interact with pIgR and, hence, does not use the transcytosis pathway. IgG (and monomeric IgA) might cross the epithelial layer using the paracellular pathway, i.e. among the epithelial cells in cases where the sealing by tight juctions is loosened^2^. IgA is the major immunoglobulin secreted by the mammary gland, parotid gland, submandibular gland, lacrimal gland and colonic mucosa^3^. CD71 (transferrin receptor 1) might function as an IgA receptor in the retrotransport of secretory IgA in complex with the gluten-derived peptides gliadins, in the active celiac disease^4^, but seems unrelated to normal processes of Ig transcytosis. Fc neonatal receptor (FcRn) is another relevant component of the transport of IgG across epithelia among other functions. FcRn binds to IgG at acidic pH and releases it at neutral pH, thereby contributing to transcytosis of IgG from the gut lumen in neonates and to the retrieval of IgG from acidic compartments after pinocytosis. More recently, FcRn has been implicated in the transfer of maternal Ig to the fetus, through the placenta^5^.

IgA and IgG are part of the many components of the prostate gland secretion^6^, and correspond to 0.1 and 0.05 mg/mL of the seminal fluid, respectively^7,8^. IgA and IgG were initially identified in association with the prostate secretion within the lumen of human prostate biopsy samples, by immunofluorescence^9^. The variation in IgA content in the prostatic fluid and serum in chronic prostatitis led to the assumption of the non-systemic character of prostate immunity^10^.

Considering the association of the prostate gland with the reproductive tract, its topography^11^, and the identification of subepithelial (stromal) IgA-rich cells in the human prostate^10^, two research groups have suggested that the prostate may be part of the *common mucosal immune system* (CMIS). Thus, after an infection episode, cells derived from the affected MALT-containing mucosae would be recruited to the prostate via specific homing. Ablin *et al*.^9^ identified IgA and IgG in association with secretory granules inside the lumen of the human prostate, and a particular association of IgG with “the basal aspect of luminal secretory cells”. These observations led the authors to suggest that epithelial cells participate in the transport of immunoglobulins to the gland lumen. However, definitive proof for the connection of the prostate gland with CMIS, the location of secreting plasma cells and the mechanism by which the immunoglobulins reach the gland lumen are unknown.

Infections of the urogenital tract and chronic prostatitis result in the presence of sIgA in the prostatic fluid^12,13^ and in higher titers of bacteria-specific antibodies in the prostate secretion as compared to the serum, in some conditions^8,14^. Connection wih the CMIS presumes the production of specfic antibodies in the absence of local infection. In addition, it is not known whether this possibility may be related to the fact that most cases of prostititis are not directly associated with the presence of an infectious agent.

Together, the available evidence reinforces the idea that the prostate gland is part of the CMIS and secretes non-specific and specific antibodies. However, current knowledge relies on results obtained from the sampling of non-uniform and/or diseased human tissues and random infections occurring locally. Definitive proof would result from direct stimulation of known CMIS organs, such as the nasal (nasal route) and intestinal mucosae (oral route), and detection of specific responses in the prostate.

Sipuleucel was designed to induce an immune response against prostate acid phosphatase and resulted in a 4.1 month improvement in the median overall survival of metastatic castration-resistant prostate cancer patients, activating dendritic cells (DC) and increasing the infiltration of CD3^+^ T cells at the tumor interface^15^. On the other hand, the oral immunostimulant OM-89 (“uro-vaxom”), an extract of heat-killed uropathogenic *E. coli*, stimulates IgG, IgA and up-regulates DC, culminating in better urinary-tract infection control^16^. However, sipuleucel and OM-89 are both still enigmatic in terms of precise mechanisms, and, in the case of sipuleucel, *in vitro* peripheral blood cell activation and reinfusion in the patient are necessary, with evident limitations regarding feasibility, costs and adverse events including chills, fever, and headache. In this scenario, immunomodulation of the prostate using the CMIS concept might represent a sophisticated, cheaper and less toxic boost of the immune system.

Herein, we tested the hypothesis that the prostate gland is part of CMIS and that epithelial cells participate actively in the transference/transport of specific immunoglobulins to the prostate secretion, which, eventually, will be part of the ejaculate. To test this hypothesis, we have (**a**) quantitated (and localized) immune system cells and the immunoglobulins IgA and IgG in the organ, (**b**) investigated whether epithelial cells were engaged in transcytosis of immunoglobulins, (**c**) identified pIgR in the prostate epithelium, and (**d**) evaluated the changes in the number and distribution of immune cells and total and antigen-specific IgA and IgG after mucosal immunization with ovalbumin.

## Results

### Immune-system cells do not organize a mucosal lymphoid tissue in the prostate gland

Immune system cells correspond to nine percent of the cells isolated by enzymatic dissociation of the rat ventral prostate (VP) (Figure S1). According to their relative abundance, these cells were mast cells (6.5%), dendritic cells (1.4%), macrophages (0.4%), CD3^+^ T cells (0.2% CD4^+^; 0.3% CD8^+^ and 0.2% TCRγδ), B cells (0.1%) and natural killer (NK) cells (0.04%) (Figures S1H,J and K).

**Figure 1:**
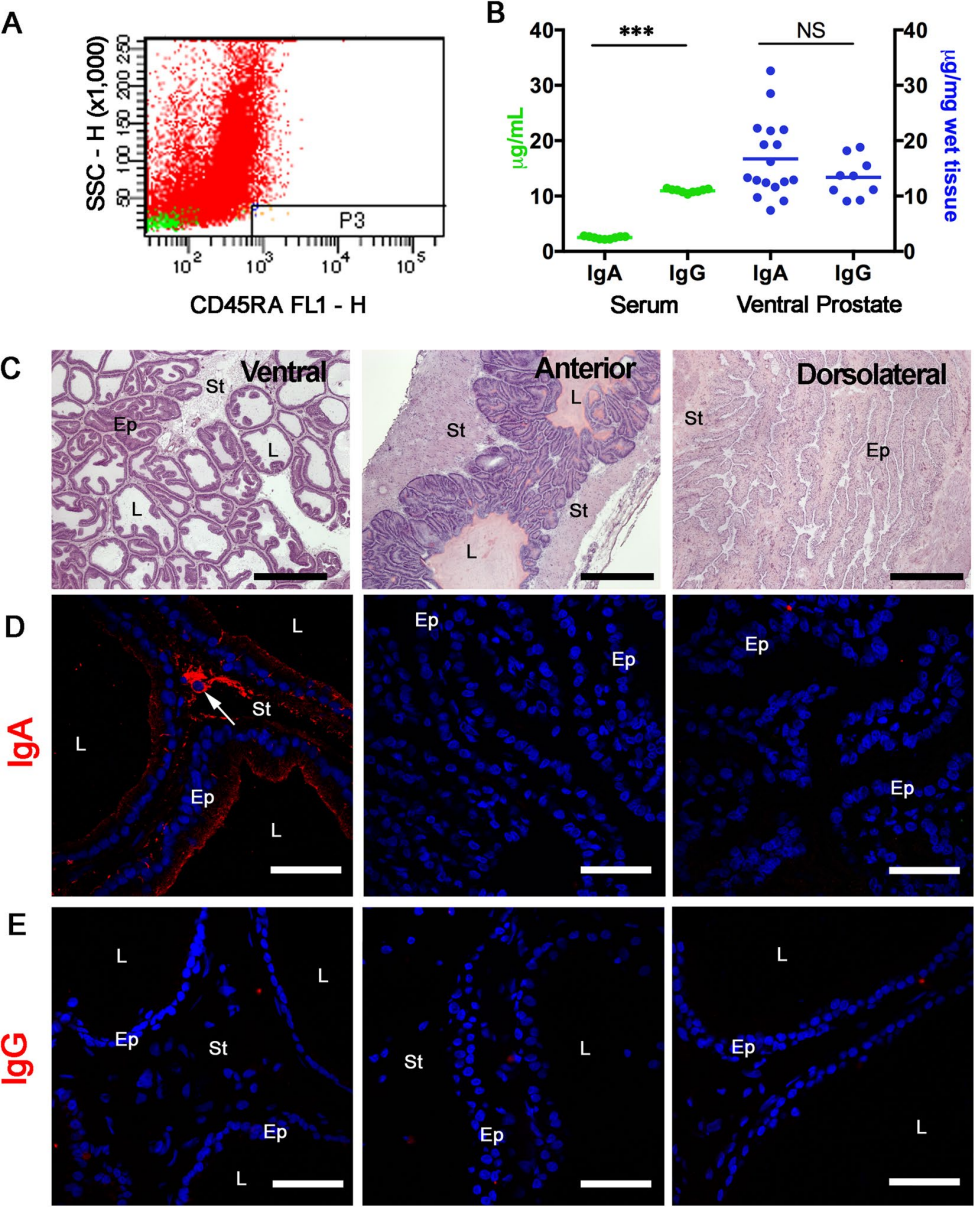
Content and distribution of immune cells and IgA in the rat prostate lobes. (**A**) Flow cytometry identified CD45RA cells as a minor fraction (0.1%) of prostate cells. (**B**) Quantification of IgA and IgG in serum and prostate extracts reveals that IgG content is about 5-fold higher in serum, while equivalent amounts of either Ig are found in the VP. (**C**) Photomicrographs of HE-stained sections of the ventral, anterior and dorsolateral prostate lobes. Immunohistochemistry and confocal microscopy were used to locate (**D**) IgA and (**E**) IgG in the ventral (VP), anterior and dorso-lateral lobes of the rat prostate. IgA was found concentrated in the apical region of the secretory luminal cells of the VP, and was absent from the anterior and dorso-lateral lobes. Few stromal cells were intensely stained for IgA (arrow). IgG staining was faint in the three prostate lobes. Nuclei appear stained blue with DAPI Ep = epithelium; St = stroma; L = lumen. Scale bars in (**C**) = 50 µm; Scale bars in (**D)** and (**E**) = 200 µm. (***p < 0.001; *t*-test).

Using histology, we identified mast cells aligned with blood vessels (Figure S1L). Immunohistochemistry revealed scattered immune cells in the stroma (Figure S2). An exhaustive search revealed no organized mucosal lymphoid tissue or epithelium-associated follicles, such as those found in other mucosae. We used immunohistochemistry to identify the same cell subsets in the dorso-lateral (DL) and anterior prostate lobes (AL). Consistently, no organized lymphoid tissue was found in the DL or in the AL, discarding the possibility that such organization could be specific to one of the prostate lobes. Indeed, it became clear that both DL and AL had fewer immune system cells than in the VP (Figure S2). To further investigate the presence of plasma cells, we performed flow cytometry and double immunohistochemistry for CD45RA and either IgA or IgG. CD45RA^+^ cells corresponded to a small population of cells in the VP in non-immunized animals (Fig. 1). Occasional IgA-rich cells were found in the stroma (Fig. 1D). IgA was localized in the luminal secretory cells in the prostate epithelium and was part of the luminal content in the VP, but not in the DL and AL (Fig. 1D). Only a few cells were positive for IgG in the three prostate lobes in non-immunized animals (Fig. 1E); double-positive IgA^+^/CD45RA^+^ plasma cells were restricted to the prostate stroma (Fig. 1D and E), and no IgG^+^/CD45RA^+^ cells were found in non-immunized rats.

### The prostate epithelium transcytoses IgA towards the gland lumen

**Figure 2:**
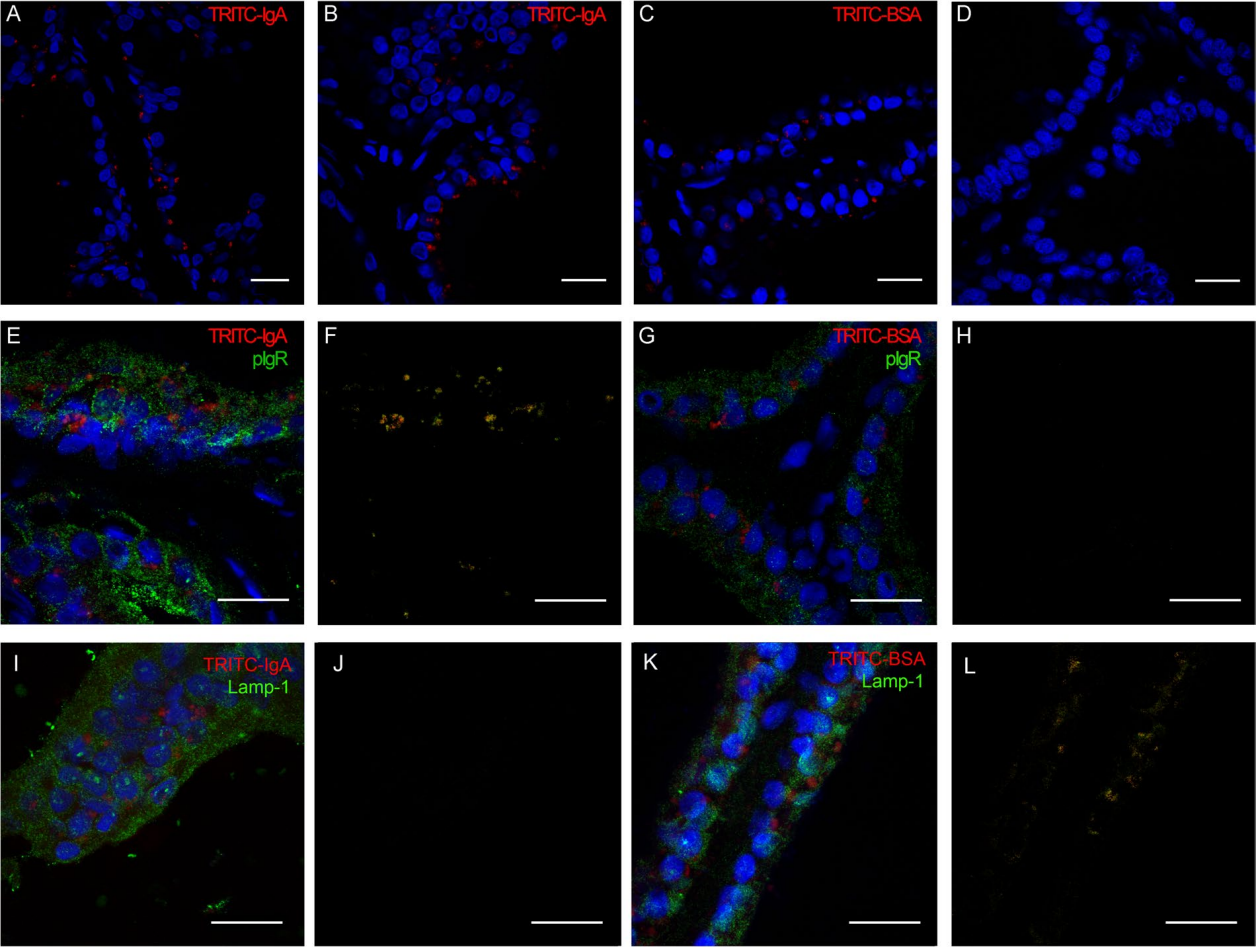
The prostate epithelium concentrates systemic soluble monomeric IgA in the secretion via transcytosis and interaction with pIgR. (**A** and **B**) TRITC-IgA was injected i.v. and monitored in the VP at different times after injection. Confocal microscopy identified the TRITC-IgA fluorescence in epithelial cells as early on as 30 min after injection (**A**), and the TRITC-IgA appeared as dot-like structures in the supranuclear region of epithelial cells and in the lumen at 6 h after injection (**B**). (**C**) TRITC-BSA was also captured by epithelial cells but was retained in the cell 6 h after injection. (**D**) Non-injected negative control. (**E** and **F**) pIgR was detected by immunohistochemistry (**E**) and colocalized with injected TRITC-IgA (**F**). (**G** and **H**) TRITC-BSA showed no co-localization with pIgR (**G**,**H**). (**I** and **J**) Lamp-1 was identified by immunohistochemistry and used as a late endosome marker (**I**). Lamp-1 did not colocalize with TRITC-IgA (**J**). (**K** and **L**) TRITC-BSA showed partial co-localization with Lamp-1 (**K** and **L**). The cell nuclei were stained blue with DAPI. Scale bars = 20 µm.

Despite the scarcity of B cells in the prostate, quantifiable and roughly equivalent amounts of IgA and IgG were found in VP extracts (Fig. 1B). Considering that IgA was found in the luminal secretory cells of the VP epithelium (Fig. 1D), we asked whether there might be a role for these cells in the transcytosis of IgA towards the gland lumen. To investigate this possibility, we injected tetramethylrhodamine-labeled IgA (TRITC-IgA) and monitored its presence in the prostate. One third of he purified IgA sample used corresponded to J-chain bound dimeric IgA (Figure S3). Thirty minutes after intravenous (i.v.) injection of TRITC-IgA, the expected fluorescence was detected, in dot-like structures at the basal region of the prostate epithelial cells (Fig. 2A). One hour after injection, we observed similar structures inside the epithelial cells and, subsequently, at the perinuclear and apical portion of the secretory luminal cells. Six hours after injection, fluorescence was found in the gland lumen (Fig. 2B). The negative non-injected control showed no fluorescence under the same conditions of image acquisition (Fig. 2D). These observations demonstrated that the prostate gland epithelium is actively involved in the transcytosis of IgA towards the gland lumen. This dotted appearance was preserved when the fluorescent material was within the gland lumen, which suggests that IgA might be secreted in membrane-bound particles, similar to prostasomes. This possibility is readly testable with the use of immunogold electron microscopy and high-resolution confocal microscopy after applying membrane-probes, but was beyond the scope of the present work.

To exclude the possibility that this was a non-specific process, we compared the co-localization of the TRITC-IgA and TRITC-labeled bovine serum albumin (BSA), admistered similarly with either polymeric immunoglobulin receptor (pIgR) or Lamp-1 (a marker for the late endosome compartment). TRITC-labeled BSA was also endocytosed by the prostate epithelium, but was eventually retained in structures similar to late endosomes or residual bodies located above the cell nucleus in the luminal secretory cells (Fig. 2C). TRITC-IgA showed excellent co-localization with pIgR, but not with Lamp-1 (Fig. 2E,F and I,J). In contrast, TRITC-BSA did not co-localize with pIgR, but showed partial co-localization with Lamp-1 (Fig. 2G,H and K,L).

### Oral immunization influences IgA and IgG concentrations in the ventral prostate

**Figure 3:**
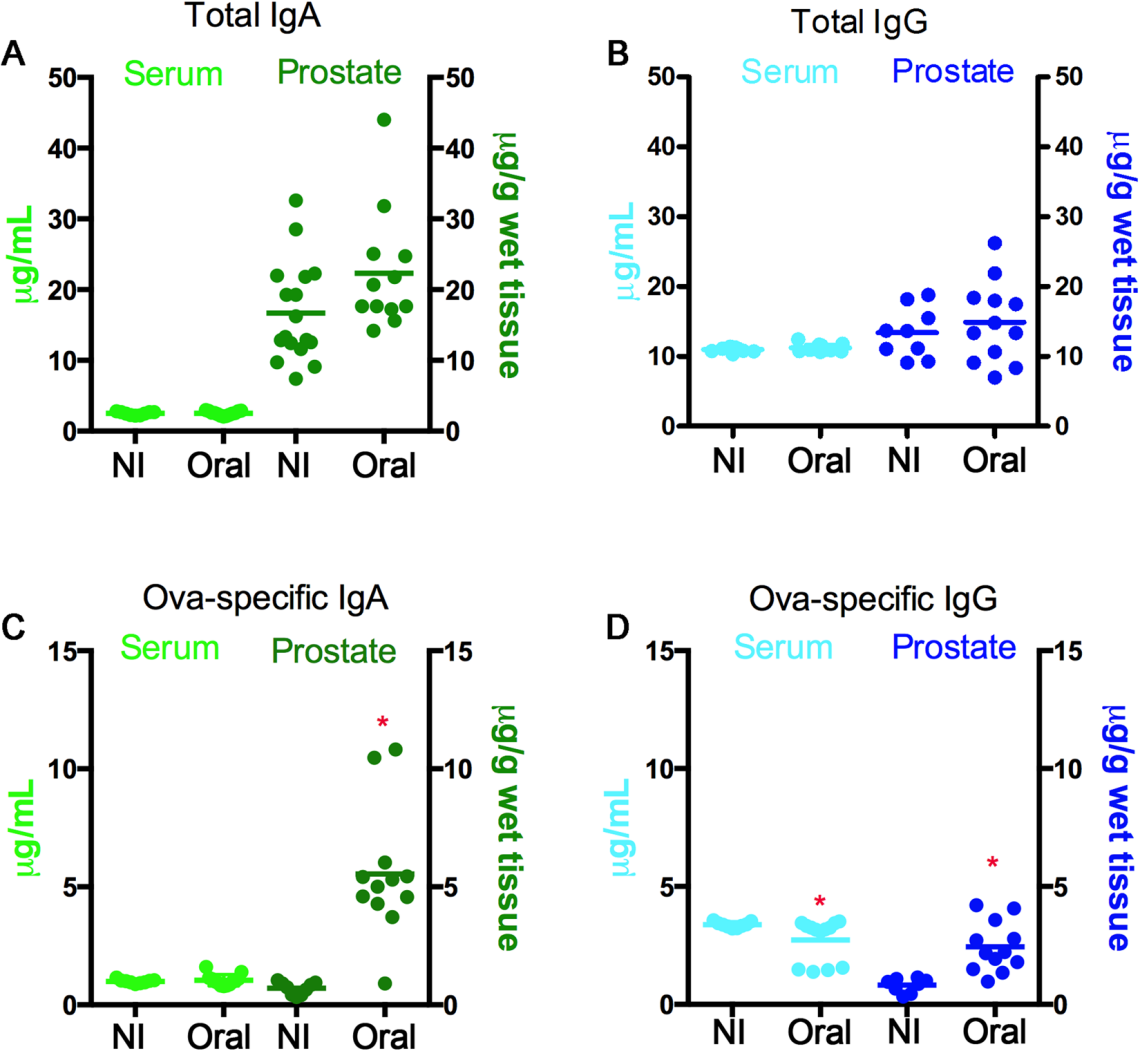
Oral immunization caused increased OVA-specific IgA and IgG in the prostate. Total (**A** and **B**) and OVA-specific IgA and IgG (**C** and **D**) were determined by ELISA in the serum and VP of control and immunized rats. Total IgA and IgG concentration was about the same in serum (**A**) and VP (**B**), before and after immunization, while IgA concentration increased by more than 10-fold in the prostate. The content of OVA-specific IgA and IgG was at baseline in the serum, but was significantly higher in the prostate (**C** and **D**), with a fairly uniform IgA response after SBA-15 administration. The asterisks indicate significant differences (*P < 0.05; *t*-test).

To determine the connection of the prostate gland with the common mucosal immune system, we immunized rats with ovalbumin (OVA), using SBA-15 as adjuvant^17^. Total and OVA-specific IgA and IgG contents were then determined in both serum and prostate by ELISA. Quantification of immunoglobulins revealed that the concentration of total IgA in the serum was approximately 2.5 µg/mL in the serum before and after oral immunization with OVA and SBA-15, while it was about 20 μg/g of wet tissue in the gland (Fig. 3A). While the small increase in total IgA was not significantly altered by immunization, the content of OVA-specific IgA showed a significant 5-fold increase (p < 0.05) in the mean content (1.1 *vs* 5.5 µg/g of wet tissue) (Fig. 3C). This is consistent with the intense staining for IgA in the gland and the observed transcytosis of systemic IgA described above.

Total IgG concentration was remarkably constant in serum and more variable in the gland, with no significant change after immunization (Fig. 3B). A fraction of these IgG was OVA-specific and immunization promoted a significant 2.5-fold increase (p < 0.05) in the content (1.0 vs 2.5 µg/g of wet tissue) (Fig. 3D) of OVA-specific IgG in the gland.

### Intraepithelial IgG^+^ plasma cells are in direct contact with the prostate lumen, while most IgA^+^ plasma cells are in the gland stroma in immunized animals

**Figure 4:**
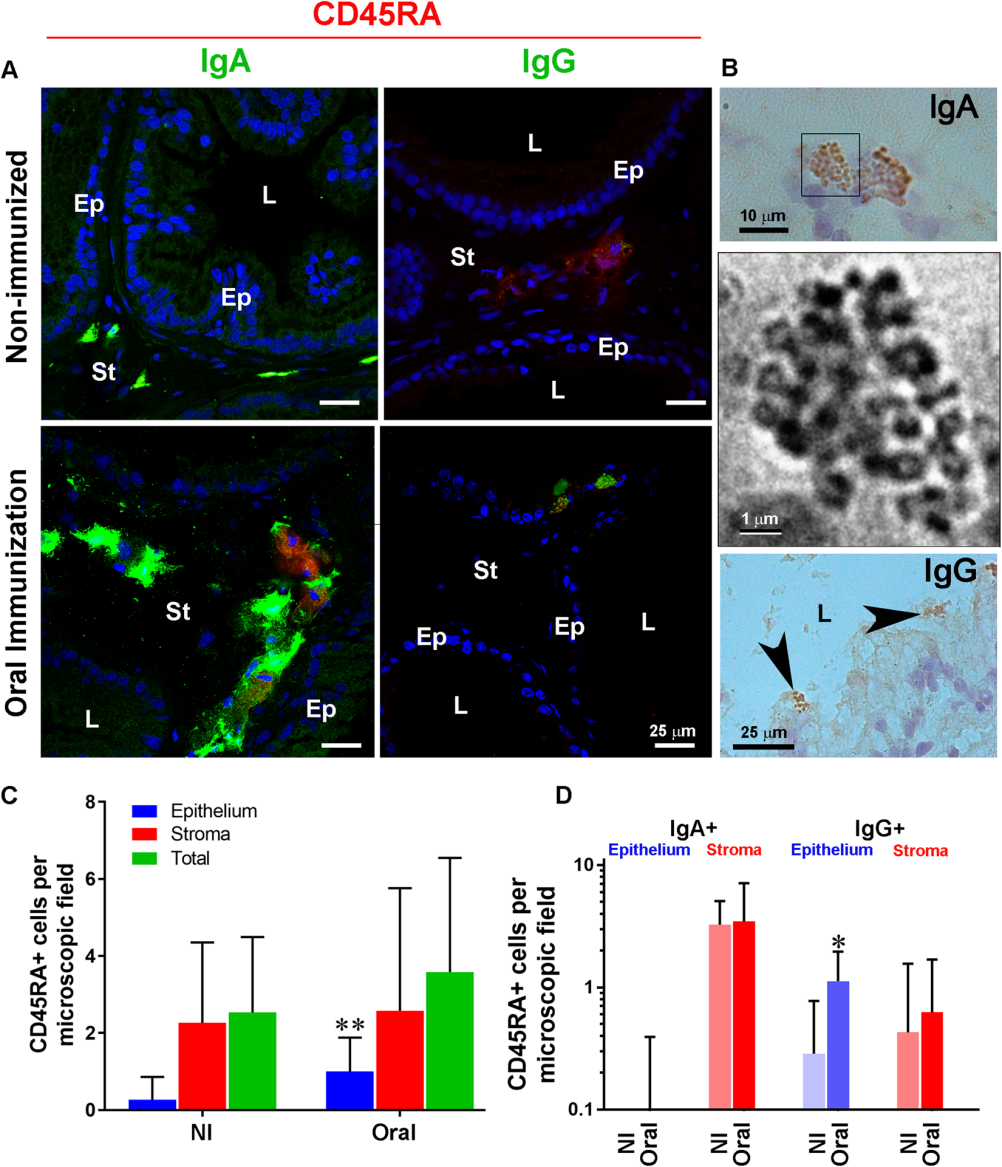
Oral immunization increases the number of CD45RA-positive cells and leads to the presence of plasma cells in the gland epithelium. (**A**) Double immunohistochemistry for CD45RA and IgA or IgG in non-immunized and immunized animals. Double positive CD45RA/IgA cells were frequent in the gland stroma of the VP in non-immunized rats. Immunization led to the appearance of double positive CD45RA/IgG in the epithelial layer. Ep = epithelium; St = stroma; L = lumen. (**B**) Immunoperoxidase staining confirmed the presence of IgA^+^ and IgG^+^ in the epithelial layer of the prostate gland. The middle panel is a contrast-enhanced detail of the enclosed area in the upper panel, showing the distribution of IgA-positive material within interconected cisternae. Scale bars in (**A**), 25 µm. Scale bars in (**B**) are 10 µm, 1 µm and 25 µm, respectively. (**C**) Quantitative results confirm that oral immunization significantly increased the number of CD45RA^+^ cells in the gland epithelium (**C**) and that most of the increment results from the increase in the number of CD45RA^+^/IgG^+^ double-positives in the epithelium. (*P < 0.05; **P < 0.01; *t*-test).

The following results suggest the existence of an active mechanism to transport antigen-specific IgA and IgG towards the prostate secretion after immunization. First, we investigated whether oral immunization affected the number of plasma cells in the gland, using double immunohistochemistry for CD45RA and IgA or IgG. A greater number of double positive IgA^+^/CD45RA^+^ plasma cells were found in the prostate stroma in the gland stroma after oral immunization (Fig. 4A). Surprisingly, we found double positive IgG^+^/CD45RA^+^ plasma cells within the gland epithelium after oral immunization (Fig. 4A). Considering that this novel finding has has not been described in the literature, we were careful to improve the resolution of Ig granules in these double-positive cells to avoid confusion with existing auto-fluorescenct granules in the epithelium, using immunoperoxidase staining (Fig. 4B). This strategy allowed not only the identification of rare plasma cells with IgA-filled granules (Fig. 3B), but also the absolute identification of cells containing IgG-filled structures within the gland epithelium, which upon high magnification and digital constrast appeared as interconnected cysterns (Fig. 3B). Additionally, the number of double-positive IgA^+^/CD45RA^+^ and IgG^+^/CD45RA^+^ in the VP of non-immunized and orally-immunized rats was quantified. The quantitative data demonstrates that oral immunization with OVA and SBA-15 increased significantly the number of CD45RA^+^ in the gland epithelium (p < 0.01) (Fig. 3C) and significantly increased the number of IgG^+^/CD45RA^+^, but not IgA^+^/CD45RA^+^, cells in the gland epithelium (p < 0.05) (Fig. 3D).

### Oral immunization using SBA-15 as adjuvant increases the dendritic cell population and modifies the cytokine profile in the ventral prostate

**Figure 5:**
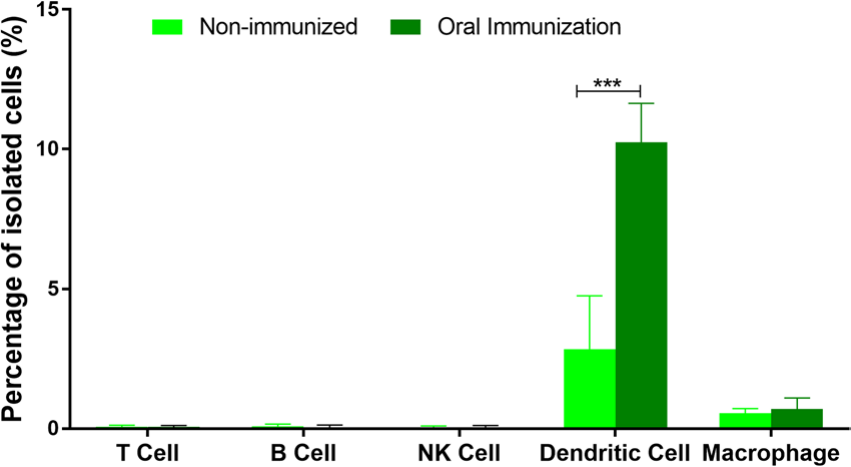
Oral immunization with OVA and SBA-15 increased the number of dendritic cells in the VP. Flow cytometry analysis of immune cell population in the VP of control and OVA-immunized animals using SBA-15 adjuvant showed a significant increase in the number of dendritic cells. The asterisks indicate significant differences. (***p < 0.001; *t*-test).

Given the good response to oral immunization using SBA-15, we also investigated whether this procedure could cause changes in the immune-cell profile in the prostate gland. Flow cytometry showed that oral immunization with SBA-15 led to an increased number of dendritic cells in the ventral prostate, while no change was observed in the number of B cells, NK cells or macrophages (Fig. 5).

**Figure 6:**
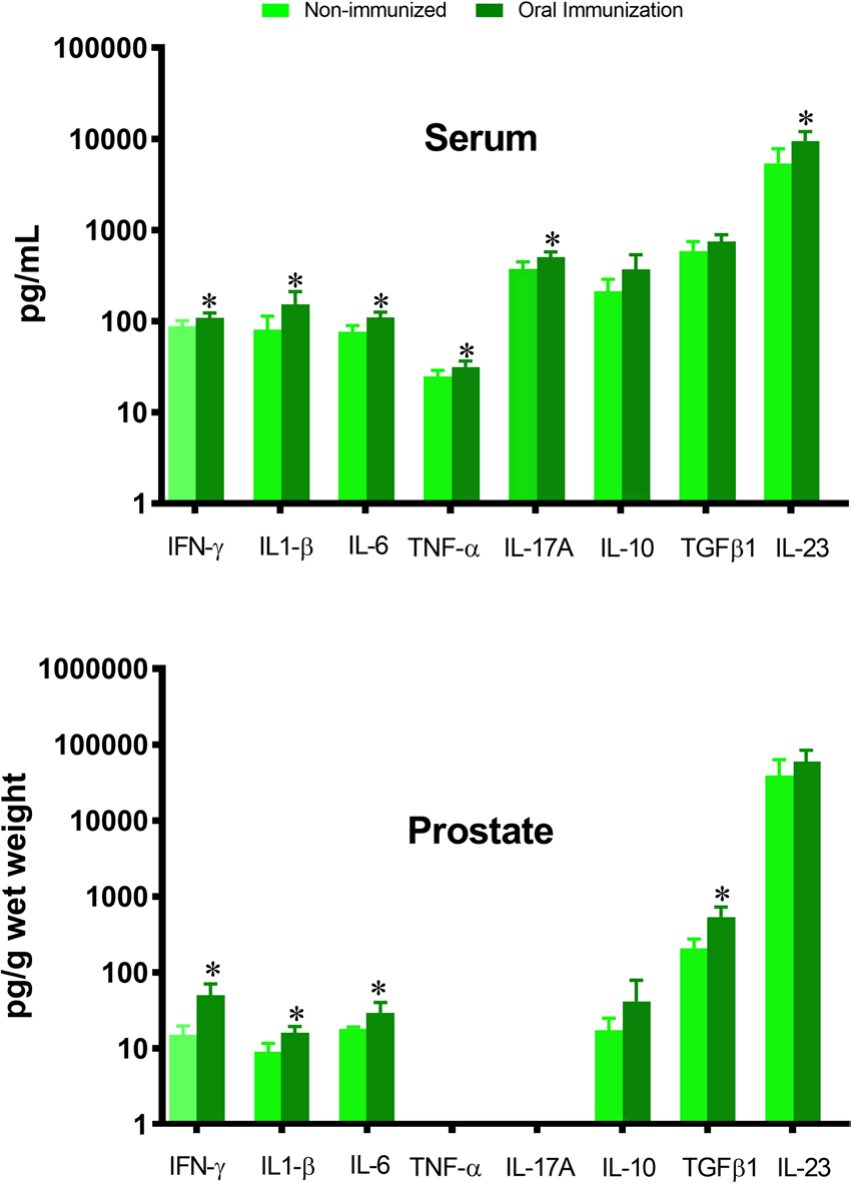
OVA-immunization using SBA-15 leads to significant changes in serum and VP cytokine profile. Cytokine profile in serum and VP of control and OVA-immunized animals using SBA-15 adjuvant, as determined by multiplex immunoassay. INF-γ, IL-6 and IL1-β concentrations increased consistently in both serum and prostate. TNF-α, IL-17A and IL-23 concentrations increased only in serum, while TGF-β increased only in the prostate. The asterisks represent differences between the control and immunized groups (*p < 0.05; Student’s *t*-test).

Due to the increased number of dendritic cells induced by oral immunization using SBA-15, we decided to extend the analyses to determine possible changes in the cytokine profile after this immunization protocol. The concentration was checked through a panel of cytokines in both the serum and the ventral prostate and found increased systemic concentrations of interferon (IFN)-γ, interleukin (IL)-1β, tumor necrosis factor (TNF)-α, IL-6, IL-17A and IL-23, while IFN-γ, IL-1β, IL-6 and transforming growth factor (TGF)-β increased locally in the gland (Fig. 6). The higher IFN-γ concentration correlated closely with the increased number of DCs in the gland.

### Nasal immunization using aluminum hydroxide affects the prostate IgA and IgG repertoire but not the dendritic cell population

**Figure 7:**
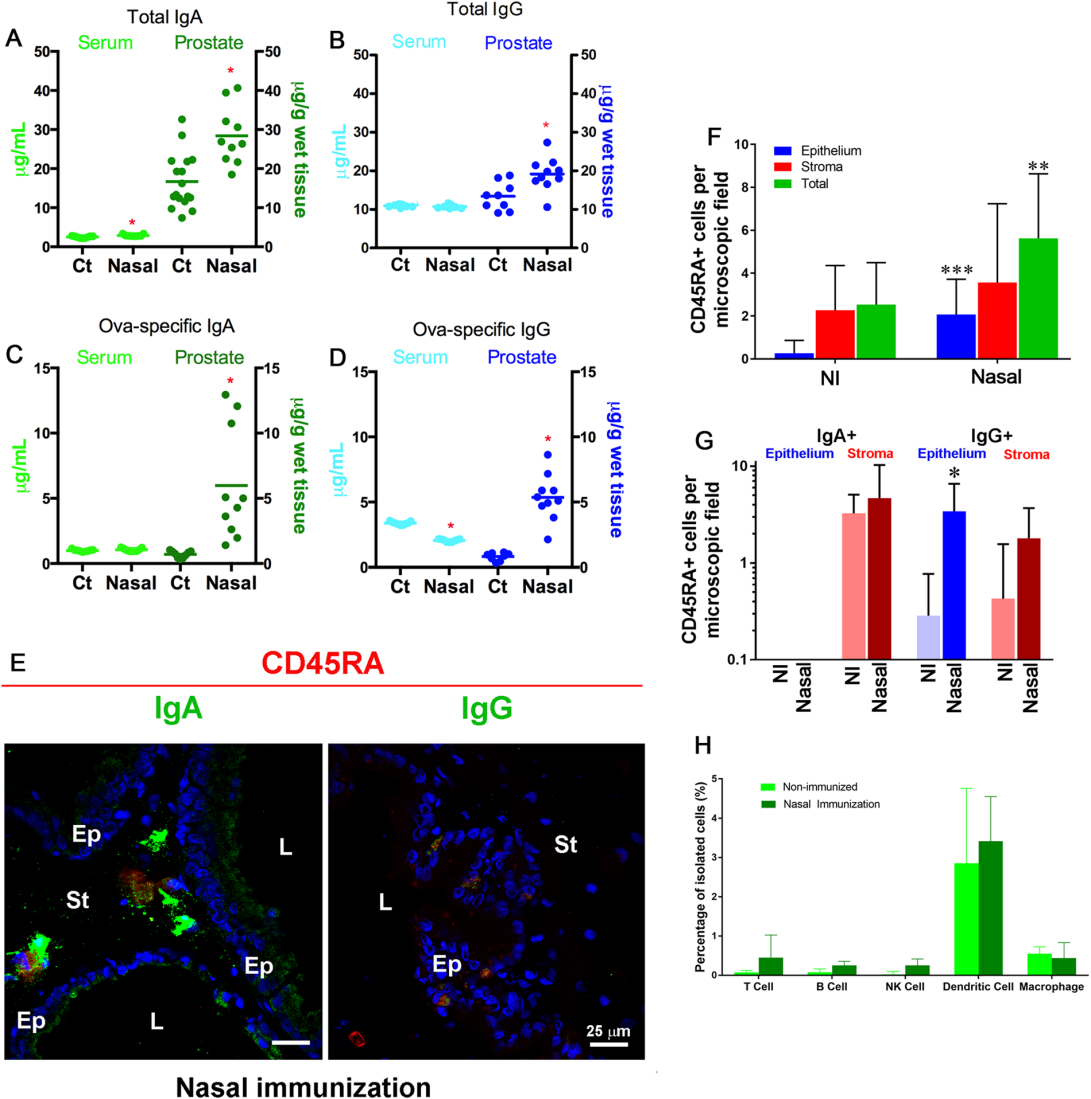
Nasal immunization using OVA and aluminum hydroxide as adjuvant affects prostate immunity. Total IgA (**A**) and IgG (**B**) contents were significantly increased after nasal immunization. The contents of OVA-specific IgA (**C**) and IgG (**D**) were also significantly increased. Double positive CD45RA^+^/IgA^+^ and CD45RA^+^/IgG^+^ were found after nasal immunization and the latter were almost exclusively found in the epithelial layer (**E**). Ep = Epithelium, St = stroma, L = Lumen; Scale bars = 25 µm. Quantitative results demonstrated an increased number of CD45RA^+^ cells in the epithelial layer (**F**) and this increase corresponded to CD45RA^+^/IgG^+^ cells (**G**). In contrast to oral immunization, nasal immunization did not affect the number of dendritic cells in the gland (**H**). (*P < 0.05; **P < 0.01; ***P < 0.001; *t*-test).

The above results demonstrate that the prostate is part of the CMIS, responding to oral immunization with OVA and SBA-15 to activate the intestinal mucosae. To further characterize the connection between the prostate gland with the CMIS, we tested whether exposure of the nasal mucosae would also promote the changes observed after exposure of the oral route/intestinal mucosae. Thus, we have immunized rats nasally with OVA and aluminum hydroxide and found that this protocol caused significant increases in total (Fig. 7A and B) and OVA-specific (Fig. 7C and D) IgA and IgG concentrations in the prostate extract. The quantification of OVA-specific IgA also showed a high variation in responsiveness (Fig. 7C). Additionally, a significant and homogenous increase was identified in the content of total IgA (Fig. 7A), in contrast with the preserved amounts of IgG in the serum (Fig. 7B). Surprisingly, the content of OVA-specific IgG dropped significantly after nasal immunization (Fig. 7D).

Due to the use of different adjuvants and routes, both types of immunization are not directly comparable, however, it is worth stressing that nasal immunization resulted in higher titers of total IgA and IgG and OVA-specific IgG. Nasal immunization also led to an increase in the number of CD45RA^+^ and IgA^+^ positive cells in the gland stroma and in the presence of CD45RA^+^/IgG^+^ cells in the prostate epithelium (Fig. 7E). The quantitative data showed a significantly increased content of CD45RA^+^ cells (p < 0.001) in the epithelium, which contributed to a change in the total number in the gland (p < 0.01) (Fig. 7F). Nasal immunization promoted an increased amount of CD45RA^+^/IgG^+^ in the gland epithelium (Fig. 7G).

In contrast to the variation in the number of plasma cells and in the content of total and OVA-specific IgA and IgG in the prostate, the number of immune cells in general and those of dendritic cells, in particular, was not affected by nasal immunization, demonstrating that exposure of the intestinal and nasal mucosae results in different profiles of prostate immunology.

## Discussion

This study demonstrates (**i**) that different cells of the immune system populate the prostate gland but do not organize a lymphoid tissue; (**ii**) that the prostate epithelium is capable of transcytosing IgA produced by stromal plasma cells towards the gland lumen in vesicles containing pIgR; (**iii**) that IgG-secreting plasma cells (and rare IgA-positive cells) are found in the gland epithelium after immunization and likely discharge IgG directly into the lumen; and (**iv**) that antigen exposure of different mucosae resulted in distinct responses of the gland, with differential effects on immunoglobulin titers, the number of dendritic cells and cytokine profiles. Jointly, the results demonstrate that the prostate is part of the CMIS.

This study confirms previous observations of IgA-positive and/or IgG-positive cells in the human prostate^9,10^ and undoubtedly identifies them as either CD45RA^+^/IgA^+^ or CD45RA^+^/IgG^+^ secretory plasma cells. Additional antibodies against more specific markers of B-lymphocytes/plasma cells used for human and mouse have been used to improve this phenotyping, but failed to identify the corresponding rat antigens.

The number of B cells in the prostate is very low, as demonstrated by flow cytometry, and the few cells found in the gland are scattered in the stroma. Despite the scarcity of B lymphocytes/plasma cells, we found that the epithelium was intensely stained for IgA (but not IgG). We have also demonstrated that intravenously-injected TRITC-IgA is actively transcytosed by the prostate epithelial cells in dot-like structures, possibly vesicles, towards the lumen. The accumulated TRITC-IgA co-localizes with pIgR, and is delivered as compact, possibly membrane-bound structures, into the lumen. Part of the injected IgA co-localizes with Lamp-1 (as TRITC-BSA does), suggesting that a small fraction of IgA is captured via non-specific endocytosis and reaches the late endosome compartments. We also observed that IgA transcytosis was restricted to the ventral lobe of the rat prostate, with evidence that the DL and AL do not perform such a role. This might correlate with a regionalization of the human prostate, with respect to the active transport of IgA to the secretion, which remains to be determined. We understand that the simple co-localization of TRITC-labelled IgA and pIgR in not a proof of direct association. However, their presence in the same compartment demonstrates that the TRITC-IgA is en route through the epithelial cells toward the lumen (i.e. undergo transcytosis). The combination of fluorescent protein conjugates and prostate spheroids in 3D matrices, and traditional biochemical analysis of the IgA complexes found in the prostate secretion will contribute to a better dissection of the mechanism of IgA transcytosis in the prostate gland.

Oral^18,19^ and nasal^2,20,21^ immunization promote an effector T and B cell-mediated immune response in female genital mucosae^18,19,22^. Hickey *et al*.^21^ showed that nasal immunization with a recombinant protein derived from a *Chlamydia’s* antigen results in the presence of antigen-specific IgA in the prostate secretion. In that study, the authors demonstrated that the levels of antigen-specific IgA were higher in the serum than in the prostate secretion.

The results presented herein demonstrate that oral administration of OVA using SBA-15 to stimulate the intestinal mucosa resulted in a uniform response, with polarization towards IgA and an increased number of local dendritic cells. The results were partially reproduced when we immunized rats with OVA and aluminum hydroxide to stimulate the nasal mucosae, which resulted in an increased number of plasma cells and higher IgA and IgG titers, but did not affect the number of dendritic cells. The IgA and IgG titers were higher after nasal immunization, but less uniform than those obtained with oral immunization.

Mucosal immunization generates a small percentage of B lymphocytes expressing peripheral homing receptors, and can result in the secretion of specific IgA and IgG to the serum^23^. We have shown an increased number of CD45RA^+^/IgA^+^ and CD45RA^+^/IgG^+^ plasma cells in the epithelial layer. Secretory plasma cells have been found in the epithelial layer of the avian oviduct^24,25^; however, this is the first time that plasma cells have been observed within the epithelial layer in a mammalian gland. It is not clear, at this time, whether these cells are in transit (as suggested for the avian oviduct), or if they reside transiently or permanently in the prostate epithelial layers. These results imply that, while most of IgA is actively transcytosed by epithelial cells, IgG is directely discharged into the gland lumen.

Mucosal immunity also predicts that mucosal dendritic cells transport the antigens to induction sites, such as lymph nodes and the spleen^23^. Intestinal dendritic cells also contribute to the production of IgA via the expression of BAFF and APRIL or RALDH2, which promotes the synthesis of retinoic acid, a known inducer of mucosal IgA^26^. This possibility aligns well with the observation that the number of dendritic cells increases in the prostate after oral immunization and correlates with a higher IFN-γ concentration, suggesting that they might have migrated directly from the intestine to the prostate, where they influence the organ immunity and secretory activity. It remains to be determined whether the increased number of dendritic cells in the VP correlates with increased levels of retinoic acid, and whether local exposure to antigens within the gland would be communicated back to other mucosae, but both these questions are beyond the more translational scope of the present investigation.

We have also shown that serum and prostate cytokine profiles changed after immunization. The increased IL-17A in serum is in close agreement with the idea that the prostate response observed is possibly a sign of an overall communication between the mucosae. IL-17A is involved in mucosal immunity, stimulating the immunoglobulin switch towards IgA^27^.

It is important to highlight that activated dendritic cells become migratory and home to adjacent lymph nodes, where they activate specific T cells. In the clinical setting, dendritic cell-based immunization induces measurable responses against the antigen, indicating that dendritic cells are promising adjuvants for antigen-specific vaccination of patients. In addition, dendritic cells are frequently used in clinical cancer treatment to induce or enhance tumor immunity, including prostate^28^ and renal^29^ cancers. It will be interesting to test whether mucosal immunization may be useful to direct the prostate immunology against cancer cells.

We believe that new immunological tools are essential steps towards the development of immunotherapies and that the present results endow us with a prototype vaccine with clear immunomodulatory potential against infectious diseases and, optimistically, against prostate cancer.

## Methods

### Animals

Adult male Wistar rats (*Rattus norvegicus albinus*) from the UNICAMP Multidisciplinary Center for Biological Investigation were used in this study. The animals were maintained in conventional housing in propylene cages, with a 12-h light-dark cycle, fed a standard laboratory diet, and given filtered water *ad libitum*. All procedures were performed in accordance with the ethical regulations established by the Brazilian College of Animal Experimentation, and were approved by the Animal Use Ethics Committee of the State University of Campinas (Protocol no. 2337-1).

### Immunization experimental design

Fifteen adult animals per group were immunized with two doses of ovalbumin (OVA; Sigma-Aldrich, Saint Louis, MO, USA) in conjunction with aluminum hydroxide (Sigma-Aldrich) adjuvant via the intranasal route, or with the mesopourous SBA-15 silica via the oral route (by gavage). The first dose was given 75 days after birth and the second, 180 days after the first i.e. when the animals were 255 days old. The animals were euthanized in a CO_2_ chamber and the samples were collected 30 days after the second immunization. In the first immunization, 4 µg OVA plus 400 µg of adjuvant were administered. In the second immunization, 40 µg OVA plus 400 µg of adjuvant were given. For the protocol using SBA-15, we used the procedure described by Carvalho *et al*.^17^. Ten control animals received only phosphate-buffered saline (PBS) + adjuvant by the same route (5 animals orally and 5 intranasally) and underwent the same process of analysis.

### Biological samples

Serum was obtained from blood by cardiac puncture and centrifuged to remove cell components. The supernatant was collected and incubated at 56 °C for 40 min to inactivate proteins of the complement system. The material was aliquoted and stored at −20 °C until used.

The prostate fluid was obtained after surgical removal of the ventral prostate and immersed in sterile PBS. The organ was then cut into very small pieces and gently homogenized. The residual tissue was collected by centrifugation and the soluble component present in the supernatant was stored at −20 °C for use in the following analyses. In each case, the content of Igs was determined relative to the total protein content.

### Enzyme-linked immunosorbent assay (ELISA)

For protein extraction, prostates from forty (fifteen per each immunized group and ten controls) rats were triturated with an electric grinder in 50 mM Tris-HCl (pH 7.4) containing 0.2 M NaCl, 0.1% Triton X-100 (Sigma-Aldrich), 10 mM CaCl_2_, and 1% protease inhibitor cocktail (Sigma-Aldrich). Total protein was quantified according to the method of Bradford^30^, using bovine serum albumin (Sigma-Aldrich) as a standard.

For quantification of total and OVA-specific IgA and IgG, a standard curve was used with different concentrations of purified IgA or IgG as a quantification parameter. The assay was performed in a 96-well flat-bottom plate and each sample was assayed in triplicate. For quantification of total IgA and IgG, each well was sensitized with 1 mg of prostate sample or serum diluted 1:1000 in 0.05 M sodium carbonate buffer, pH 9.6 in a final volume of 100 μl per well. After 3 h at 37 °C, non-specific binding sites were blocked with 3% BSA (Sigma-Aldrich) during 1 h at 37 °C and incubated overnight at 4 °C with biotinylated rabbit anti-IgA Fc or rabbit anti-IgG Fc, (Rheabiotech, Campinas, SP, Brazil). After repeated washings with PBS containing 0.5% Tween-20 (PBS-T) (Sigma-Aldrich), wells were incubated with streptavidin peroxidase conjugate (Sigma-Aldrich) diluted 1:1000 in PBS for 1 h at 37 *°*C. Finally, after washing, the reaction was continued with the addition of 0.6 mg/mL O-phenylenediaminedihydrochloride (OPD; Sigma-Aldrich) in 0.1 M citrate-phosphate buffer pH 5.6 at room temperature for 1 h. The peroxidase activity was blocked with 2 N H_2_SO_4_ and the optical density (OD) measured in a spectrophotometer at λ = 492 nm.

For quantification of OVA-specific IgA and IgG, the plates were sensitized with 2 mg/mL of OVA in 0.05 M sodium carbonate buffer pH 9.6 in a final volume of 100 µl per well for 4 h at 37 °C. After three washes with PBS -T the wells were incubated with 3% BSA for 1 h at 37°C to prevent nonspecific binding of proteins, and then incubated with PBS-diluted prostate and serum biological samples, overnight at 4 °C. After repeated washing in PBS-T, the protocol used was identical to that described above.

Concentrations of Igs were expressed relative to total protein content in the specimens. In the case of prostate fluid, Igs concentrations were expressed relative to the prostate weight of each animal.

Cytokine protein levels were quantified using Luminex’s xMAP Technology with Milliplex kits (Millipore, Billerica, MA, USA), according to the manufacturer’s instructions. In brief, 15 µg of total protein per sample were mixed with the conjugated antibody capture beads, before adding the phycoerythrin-streptavidin-conjugated reporter antibody. The double-labeled beads were separated and quantified in a Luminex xMAP flow cytometer. The experiments were carried out in triplicate.

### Ventral prostate cell isolation and flow cytometry

Ventral prostate cells from thirty (ten per each immunized group and ten controls) rats were isolated by sequential treatment with collagenase and trypsin, before processing with specific antibodies to quantify immune-cell types by flow cytometry analysis.

Immediately after removal, the ventral prostate from each animal was minced in Earle’s balanced salt-solution (EBSS) (Life Technologies Corporation, Grand Island, NY, USA) containing 1% sterile antibiotic. After 3 washes with EBSS, small fragments were placed in a tube containing 5 mL of 1 mg/mL collagenase type I (Life Technologies) in EBSS and incubated at 37 °C for approximately 3 h under constant and vigorous agitation. After centrifuging at 1500 rpm for 10 min, the collagenase solution was removed and the cells resuspended in 5 mL of 0.25% trypsin (Sigma-Aldrich) and incubated for 20 min at 37 °C. Trypsin activity was inhibited by adding 10 mL of EBSS supplemented with 10% fetal bovine serum (FBS; Sigma-Aldrich), and after centrifuging at 1,500 rpm for 10 min the pelleted cells were resuspended in sterile PBS containing 2% FBS.

After counting in a hemocytometer, 10^6^ cells were used in each labeling reaction. Markers for macrophages (mouse anti-CD68 conjugated to biotin plus APC-streptavidin conjugatedand mouse anti-CD163 PE-conjugated), NK cells (mouse anti-CD161aPE-conjugated), B lymphocytes (mouse anti-CD45RA FITC-conjugated), T lymphocyte TCRαβ (APC-conjugated mouse anti-CD3, PE-conjugated mouse anti-CD4, FITC-conjugated mouse anti-CD8), T lymphocyteTCRγδ (APC-conjugated mouse anti-CD3 and PE-conjugated mouse anti-TCRγδ), dendritic cells (mouse anti-CD103 plus Alexa Fluor 488-conjugated goat anti-mouse Ig [Invitrogen, Carlsbad, CA, USA]), mast cells (rabbit anti-tryptase [Santa Cruz Biotechnology, Dallas, TX, USA] plus Alexa Fluor 546-conjugated goat anti-rabbit antibody [Invitrogen]) all obtained commercially from BD Biosciences (Pharmingen, San Diego, CA, USA) unless otherwise mentioned and used according to the manufacturer’s instructions and recommended concentrations.

After labeling, cells were fixed with 4% paraformaldehyde, quantified in a BD FACSCalibur flow cytometer, and analyzed with BD FACSDiva software (BD Biosciences, San Jose, CA, USA).

Before tryptase labeling to identify mast cells, the cells were fixed in 4% paraformaldehyde (Sigma-Aldrich) for 20 minutes followed by membrane permeabilization in PBS-T for 1 h.

Negative controls were made for each panel of markers using the same prostate cell preparation without the addition of antibodies conjugated to fluorophores. In the case of dendritic cells and mast cells, which were indirectly labeled, besides the above described control. After acquiring 10,000 events in the P1 gate, negative control tubes were analyzed for each filter to exclude endogenous fluorescence and the same parameters used in negative controls were used for subsequent analyzes (Fig. 1). Analyzes were performed based on the acquired events within the P1 gate (or P3 for T cells) and the results refer to the percentage of positive cells for each specific marker in relation to the total number of events acquired independently.

### TRITC-IgA and TRITC-BSA administration and imaging

Rat IgA purified from ascites (Invitrogen) was used in this experiment. The IgA sample contained a 2:1 monomer:dimer ratio. The presence of J-chain was confirmed by SDS-PAGE (Figure S3) and mass spectroscopty (MS). J-chain corresponded to about 3% of the total IgA (H + L + J chains) mass. MS revealed 7 peptides out of the 12 predicted trypsin-digestion products were found, covering 60% of the primary sequence. MS has also identified that the light chain was the kappa-type (7 peptides out of 10 predicted trypsin-digestion products).

IgA and BSA were conjugated with TRITC (Sigma-Aldrich) by incubation in 100 mM carbonate/bicarbonate buffer, pH 9.0. Excess free TRITC was removed by extensive dyalisis against the same buffer and then against PBS, in the dark. TRITC-IgA or TRITC-BSA conjugates 50 μg were injected intravenously through the rat tail vein. The animals were euthanized after 0.5, 1, 1.5, 6, 8 and 24 h. and their prostates were removed and placed in OCT mounting medium (Tissue Tek; Sakura Finetek, Torrance, CA, USA). Cryostat sections (7 µm thick) were fixed for 15 min with methanol at −20 °C and the nuclei were stained with 4′,6-diamidino-2-phenylindole (DAPI) (Sigma-Aldrich). The sections were mounted in 90% glycerol in water. Fluorescence was evaluated under a LSM780 confocal microscope (Carl Zeiss, Jena, Germany).

Further analysis of co-localization of IgA-TRITC and BSA-TRITC injected at all times described with pIgR or Lamp-1 were performed using immunofluorescence for both markers. Analyses were done using the Zen 2010 software (Carl Zeiss) and demonstrated by the mask image showing the co-location of the red (TRITC-IgA or TRITC-BSA) and green fluorescence (pIgR or Lamp-1).

### Immunohistochemistry

Five prostates from each experimental group were dissected and immersed in OCT mounting medium (Tissue Tek), serial cryostat sections (7 µm thick) were fixed for 15 min with methanol at −20 °C, stained with hematoxylin and eosin or immunoperoxidase or immunofluorescence.

For the immunoperoxidase or immunofluorescence, nonspecific binding sites of the sections were blocked with 3% BSA diluted in PBS for 1 h. The specimens were then incubated with rabbit anti-pIgR (Novus Biologicals, Littleton, CO, USA) diluted 1:1000, rabbit anti-Lamp-1 (Novus Biologicals) diluted 1:1000, mouse anti-CD68 (Millipore) diluted 1:500, rabbit anti-CD3 (Abcam Inc., Cambridge, MA, USA) diluted 1:100, mouse anti-CD79A (Sigma-Aldrich) diluted 1:1000, mouse anti-CD103 (BD Biosciences Pharmigen) diluted 1:500, mouse anti-CD45RA (BD Biosciences Pharmigen) diluted 1:200, rabbit anti-IgA Fc portion (Rheabiotech) diluted 1:1000 and rabbit anti-IgG Fc portion (Rheabiotech) diluted 1:500 in PBS containing 1% BSA, incubated overnight at 4 °C.

After repeated washings with PBS containing 0.1% Tween 20, for immunofluorescence staining, sections were incubated with Alexa Fluor 546-conjugated goat anti-rabbit Ig (Invitrogen) diluted 1:2000 or Alexa Fluor 488-conjugated goat anti-mouse Ig (Invitrogen) diluted 1:1000 or Alexa Fluor 488-conjugated donkey anti-rabbit Ig (Abcam) diluted 1:1000. DAPI (Sigma-Aldrich) was used to counterstain the cell nuclei. The slides were mounted in 90% glycerol in water. The images were evaluated under a LSM780 confocal microscope (Carl Zeiss). For immunoperoxidase staining, sections were incubated with Novolink Polymer Detection Systems (Leica Biosystems, Newcastle, United Kingdom) for the visualization of mouse IgG, mouse IgM and rabbit IgG. Color development was carried out substrate/chromogen, 3,3′-diaminobenzidine (DAB), prepared from DAB chromogen and Novolink DAB Substrate Buffer (Leica Biosystems). Reaction with the peroxidase produces a visible brown precipitate at the antigen site. Sections were counterstained with Hematoxylin and coverslipped. The images were evaluated under an Axio observer Z1 light microscope (Carl Zeiss). Controls consisted of reactions from which the primary antibody incubation step was omitted.

For quantification CD45RA^+^/IgA^+^ and CD45RA^+^/IgG^+^ cells, ten microscopic fields were assessed for each animal. Based on the counts, the relative values of cellular profiles per stromal and epithelial area were attained.

### Statistical Analyses

Student’s *t*-test was used to compare the means obtained for different kinds of immune cells in the flow cytometry experiment, differences relative to the IgA/IgG contents and, the cytokine concentration data from non-immunized and each immunized group. Tests were performed with GraphPad Prism 5.0 software (GraphPad Inc., San Diego, CA, USA). *p* < 0.05 was considered statistically significant and smaller *p* values are presented.

## Electronic supplementary material

Supplementary information
