# Supplementary material for: The origin of prostate gland-secreted IgA and IgG
Source: Sci Rep. 2017 Nov 28;7:16488. doi: 10.1038/s41598-017-16717-3 (PMC5705656; doi:10.1038/s41598-017-16717-3)
Supplement: Supplementary file 1 — Supplementary information [file 41598_2017_16717_MOESM1_ESM.pdf]

Supplementary Information

The origin of prostate gland-secreted IgA and IgG

Juliete A. F. Silva, Manoel F. Biancardi, Dagmar R. Stach-Machado, Leonardo O. Reis, Osvaldo A. Sant'Anna and Hernandes F. Carvalho

Supplementary Figures

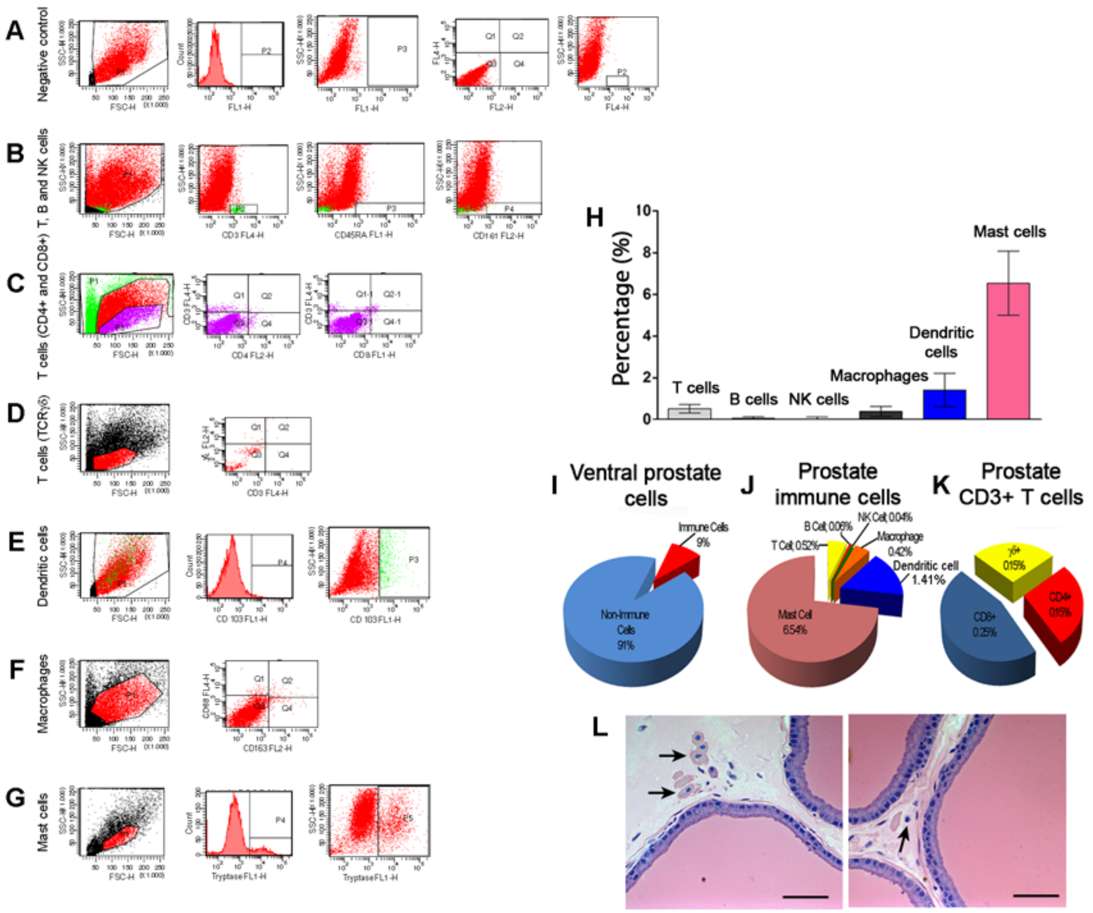

Figure S1: **Immune system cells in the prostate**. Flow cytometry was used to quantify immune cells in the prostate. Ten thousand events were acquired on the size graph (forward scatter - FSC-H) versus cellular complexity (side scatter - SSC-H) inside gate P1 (**A, B, D, E, F** and **G**). In (**C**), 10,000 events were counted in gate P3. (**A-G**) are representative dot plots of flow cytometry analysis from a single non-immunized animal. (**A**) The figure shows the gate strategy from the negative controls excluding the endogenous fluorescence and those that received only the secondary antibody used to identify mast cells and dendritic cells in the gates P2 and P3 and quadrants Q1, Q2 and Q4 for each filter (FL1, FL2 and FL4). (**B**) Gates P2, P3 and P4 show the CD3, CD45RA and CD161a positive cells corresponding to T- and B-lymphocytes, and NK cells, respectively. (**C**) Double labeling for CD3/CD4 is seen in quadrant Q2 and CD3/CD8 seen in Q2-1. (**D**) Double labeling for CD3/TCRγδ is seen in quadrant Q2. (**E**) Gates P3 and P4 show CD103+ cells. (**F**) Quadrants Q1 and Q2 showed CD68+ cells, Q2 and Q4 show CD163+ cells and Q2 shows the CD68/CD163 double positives. (**G**) P4 and P5 gates show tryptase-positive cells. (**H**) Quantitative results (mean and standard deviation) for each immune cell type presented as the percentage of the total cells isolated enzymatically from the VP. (**I**) The immune system cells corresponded to 9% of the VP cells and (**J**) were distributed in major cell types. (**K**) T cells ($CD3^+$; 0.5%) were distributed in three major classes: $CD3^+/CD4^+$, $CD3^+/CD8^+$ and CD3+/γδ. (**L**) Photomicrographs of HE-stained VP sections showing the location of mast cells near blood vessels (arrows). Scale bars in (L) = 50 μm.

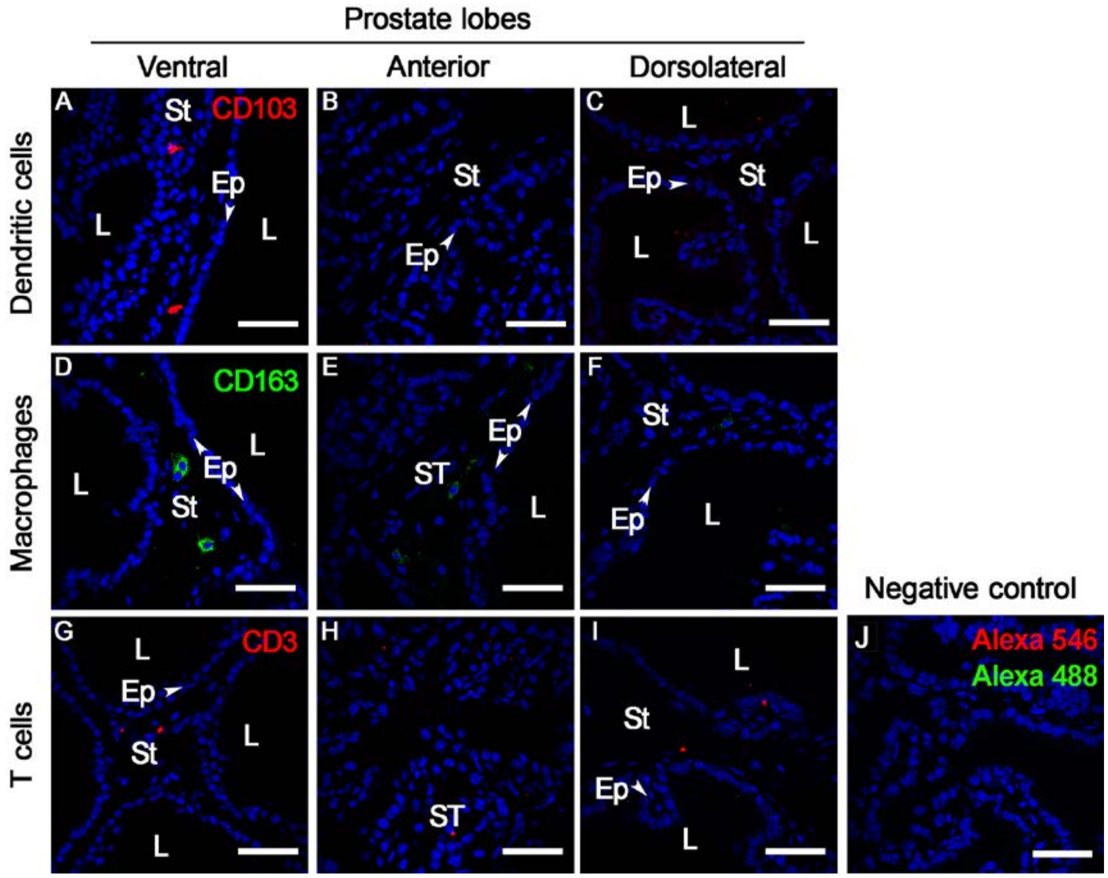

Figure S2: **Identification and localization of immune cells in the rat prostate lobes**. Immuno-histochemistry and confocal microscopy were used to locate (**A-C**) dendritic cells (CD103); (**D-F**) macrophages (CD163-ED2), and (**G-I**) T cells (CD3); in the ventral (VP), anterior and dorso-lateral lobes of the rat prostate. Immune cells were virtually absent from the anterior and dorso-lateral lobes. Nuclei were stained blue with DAPI. (**J**) The negative controls of the immunofluorescence consisted of specimens from which the primary antibody incubation step was omitted. Ep = epithelium; St = stroma; L = lumen. Scale bars = 50 μm.

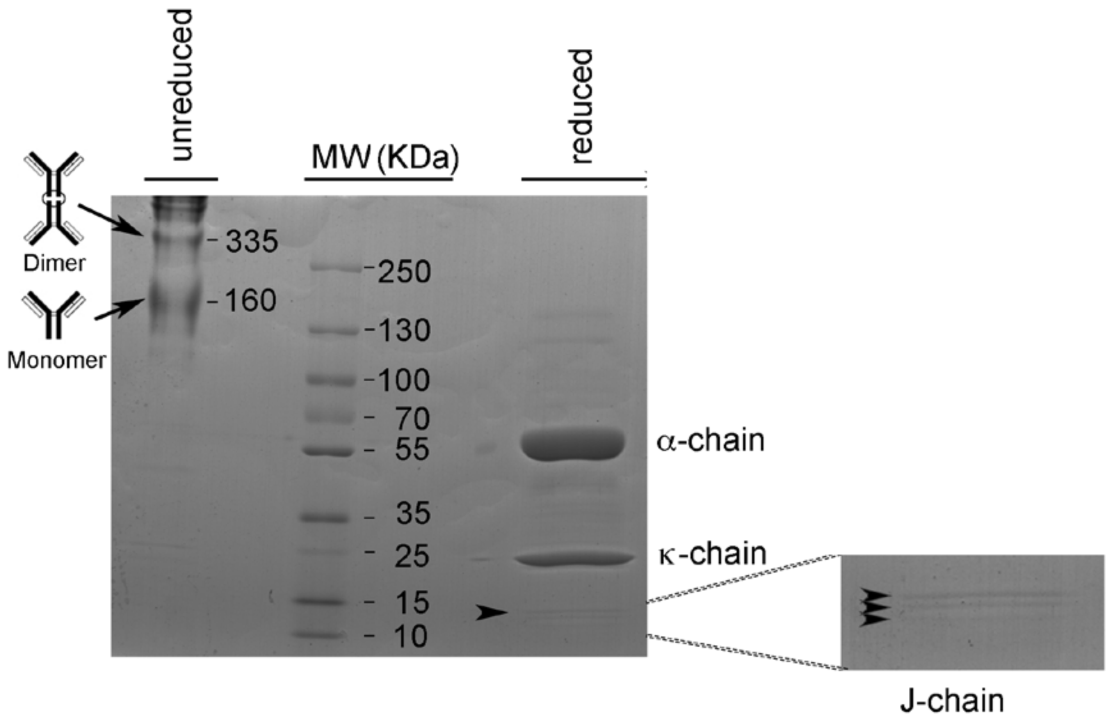

Figure S3: Characterization of the rat IgA by SDS-PAGE. A 4-20% gradient gel was used. Unreduced and reduced (0.5% mercaptoethanol) samples were electrophoresed and stained with Coomassie Blue R250. The unreduced sample contained high molecular mass complexes. IgA monomer (160 KDa) and dimers (335 KDa) are indicated. Densitometry of the corresponding bands revealed a 2:1 monomer:dimer ratio. The reduced sample showed two predominant bands corresponding to the IgA alpha (55 KDa) chain and kappa-chain (25 KDa). MS revealed the identity of the light chain. The J-chain appeared as three bands migrating at about 15 KDa in the reduced sample. Densitometry showed the J-chain to correspond to about 3% of the total IgA mass. This is a good estimation, given that the J-chain connects the IgA dimer (one third of the IgA, measured in the non-reduced sample) and about 8% of the calculated mass of the dimer (H+L+J chains).
